# Lateral optical confinement of GaN-based VCSEL using an atomically smooth monolithic curved mirror

**DOI:** 10.1038/s41598-018-28418-6

**Published:** 2018-07-09

**Authors:** Tatsushi Hamaguchi, Masayuki Tanaka, Jugo Mitomo, Hiroshi Nakajima, Masamichi Ito, Maho Ohara, Noriko Kobayashi, Kentaro Fujii, Hideki Watanabe, Susumu Satou, Rintaro Koda, Hironobu Narui

**Affiliations:** Compound Semiconductor Development Department, Device Technology Development Division, Sony Corporation, Atsugi, Kanagawa 243-0014 Japan

## Abstract

We demonstrate the lateral optical confinement of GaN-based vertical-cavity surface-emitting lasers (GaN-VCSELs) with a cavity containing a curved mirror that is formed monolithically on a GaN wafer. The output wavelength of the devices is 441–455 nm. The threshold current is 40 mA (*J*_th_ = 141 kA/cm^2^) under pulsed current injection (*W*_p_ = 100 ns; duty = 0.2%) at room temperature. We confirm the lateral optical confinement by recording near-field images and investigating the dependence of threshold current on aperture size. The beam profile can be fitted with a Gaussian having a theoretical standard deviation of *σ* = 0.723 µm, which is significantly smaller than previously reported values for GaN-VCSELs with plane mirrors. Lateral optical confinement with this structure theoretically allows aperture miniaturization to the diffraction limit, resulting in threshold currents far lower than sub-milliamperes. The proposed structure enabled GaN-based VCSELs to be constructed with cavities as long as 28.3 µm, which greatly simplifies the fabrication process owing to longitudinal mode spacings of less than a few nanometers and should help the implementation of these devices in practice.

## Introduction

GaN-based vertical-cavity surface-emitting lasers (GaN-VCSELs) are attracting interest because of their properties: low threshold current, arraying capability, and applicability to high-frequency operation. These advantages offer GaN-VCSELs significant potential as light sources for optical storage, laser printers, projectors, displays, solid-state lighting, optical communications, biosensors, and many other applications. However, GaN-VCSELs have not yet been commercialized. The main reason for this seems to be the difficulty in fabricating bottom mirrors and the absence of a method for lateral optical confinement. This article presents a new approach, introduction of a curved mirror, to resolve these two problems simultaneously.

Distributed Bragg reflectors (DBRs) made of epitaxially grown GaN-based semiconductors have been of great interest for decades. Although there have been reports of using Al(Ga)N/GaN pairs for DBR, a limited number of studies^1–4^ have reported a reflectivity of more than 90%, owing to cracks caused by the lattice mismatch between Al(Ga)N and GaN. AlInN, which has a lattice constant comparable to that of GaN at an Al content of about 80–85%, is another candidate for forming GaN-based semiconductor DBRs. Crack-free AlInN/GaN DBRs have been reported to exhibit a reflectivity close to 100% (e.g., 99.7% for Al0.82In0.18 N/GaN), and the latest study demonstrates the continuous wave operation of a GaN-VCSEL using an AlInN/GaN DBR for the bottom of the device^5^. However, the narrow stopband of AlInN/GaN remains a drawback. For example, a 50-pair Al0.82In0.18N/GaN DBR has a stopband width of 30 nm at a reflectivity of 50%, which shrinks to several nanometers at 99.5%. This necessitates precise control over the growth rate of GaN and AlInN at sub-percentage levels in order to keep stop band subsume the cavity mode of the device. Such precision is still challenging even in laboratories.

The adoption of dielectric materials seems to offer a pragmatic approach to constructing DBRs without such manufacturing concerns, considering they can possess stopbands 4–5 times wider than those of semiconductors. Even GaN-VCSELs, which use semiconductor DBRs for their bottom mirrors, utilize dielectric DBR mirrors as their top mirrors. Higuchi *et al*. reported GaN-VCSELs with DBRs, composed of dielectric materials, on both the top and bottom sides^6^. Before deposition of the bottom DBR, they bonded the top side of the wafer, which featured a prefabricated top DBR made of dielectric materials, to a silicon plate and lapped the bottom side to leave layers comprising the top DBR, p-GaN, InGaN quantum wells, and n-GaN. Subsequently, a dielectric DBR was deposited on n-GaN for the bottom mirrors.

**Figure 1:**
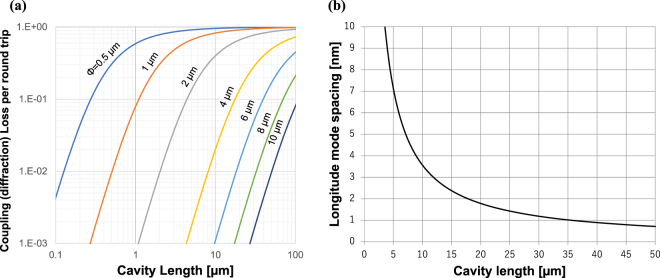
(**a**) Calculated coupling (diffraction) losses per round trip for beams having Gaussian lateral profiles with a beam waist from 1 µm to 10 µm. (**b**) Longitudinal mode spacing calculated for the following parameters: n = 2.45, λ = 450 nm, dn/dλ = −0.001.

However, such GaN-VCSELs face another challenge, namely, difficulty in controlling the cavity length. Generally, the cavity must simultaneously meet two conditions to allow laser operation. The first condition concerns the optical mode loss. Figure 1(a,b) plot the roughly estimated coupling (diffraction) loss and mode spacing as a function of cavity length (see details of the calculation in the Methods section). In Fig. 1(a), a shorter cavity is clearly better because it leads to smaller coupling (diffraction) loss after a round trip. Accounting for the fact that the estimated amplification rate for VCSELs is about 1%^7^, the cavity should preferably be shorter than several microns to limit the diffraction loss to sufficiently less than 1%. The second condition concerns the longitudinal modes. The wavelength of each longitudinal mode is determined from the cavity length, which must be controlled so that one of the modes overlaps with the gain spectrum of the active region used in the device. Even though the loss seems to be low enough for, e.g., a 5-μm-long cavity, the mode spacing of such a short cavity is as wide as roughly 7 nm (see Fig. 1(b)), where the modes easily miss the gain spectrum and fail in lasing unless the cavity length is precisely controlled to allow one of the longitudinal modes to hit near the top of the gain spectrum of the InGaN quantum wells. One can easily imagine that highly sophisticated technology would be required to control the cavity length to below several microns by polishing. Longer cavities can provide narrower longitudinal mode spacing to help one of the modes to hit near the top of gain spectrum without precise control of the cavity length. For example, a cavity longer than 20 µm gives rise to a longitudinal mode spacing narrower than 2 nm, which is sufficiently narrow to overlap the gain spectrum of typical InGaN quantum wells. However, such a long cavity often provides diffraction loss of greater than 1%, which is large enough to prevent the device from lasing. These requirements for cavity length has prevented the mass production of GaN-VCSELs.

Apart from the approach reported by Higuchi *et al*.^6^, in which a lapping process is used to provide a basal plane for dielectric DBR deposition, there are remarkable approaches for fabricating GaN-VCSELs with dielectric DBRs. For example, using a thinning process with photelectrochemical (PEC) etching^8^ and cavity formation using epitaxial lateral overgrowth (ELO) of n-GaN^7^ resulted in GaN-VCSEL laser operation for a short cavity of 1.2 µm and 4 µm, respectively. If those approaches allow precise control of cavity length, they could enable mass production of GaN-VCSELs. However, for those approaches, there are limited reports from a few organizations and no article has thoroughly illustrated results endorsing precise cavity length control (i.e. stability of lasing wavelength over a wafer).

One approach to overcoming this hurdle is the introduction of lateral optical confinement. There have been few studies on this topic for GaN-VCSELs. Hashemi *et al*. simulated the lateral optical confinement effect of step-like lateral structures on GaN-VCSELs internal loss^9^. Hayashi *et al*.^10^ experimentally demonstrated one of those structures and showed the multi-lateral-mode of the device as evidence for the occurrence of lateral optical guiding. However, step-like lateral structures essentially induce scattering loss. In the present paper, the authors propose a GaN-VCSEL having a cavity with graded lateral structures instead of a mesa structure. Resonators cladded with curved and plane mirrors are known to form stable cavities without diffraction and scattering loss^11^, exhibiting a beam waist on the plane mirror. Thus, the introduction of curved mirrors on one side of VCSELs may allow for longer cavities. This would help relax the process requirements for polishing, which have prevented GaN-VCSELs from reaching mass production. Though a long cavity could have adverse effects on some VCSELs merits, such as single longitudinal mode operation, several of the other benefits, such as a low threshold current due to the small volume of the active region, arraying capability and simple fabrication process, should be still available with such a structure.

Previous research on GaN-VCSELs has avoided apertures of less than several microns in diameter^6–8,10,12–18^, seemingly to suppress diffraction loss. Achieving lateral optical confinement could lead to smaller current apertures than ever before. The beam shape formed on the plane mirror in this type of cavity can be calculated using the following formula based on classical Gaussian optics^19^,

1 $${\rm{\sigma }}=\frac{1}{2}\sqrt{\frac{\lambda }{n\pi }\sqrt{LR-{L}^{2}}},$$

where σ is the standard deviation of the Gaussian profile, n is the equivalent refractive index of the cavity medium, L is the cavity length, and R is the radius of curvature of the curved mirrors. Thus formula predict a beam waist can be controlled by L and R of a cavity. Theoretically, the lateral optical mode can be shrunk to the diffraction limit, giving a very small aperture and small threshold currents.

**Figure 2:**
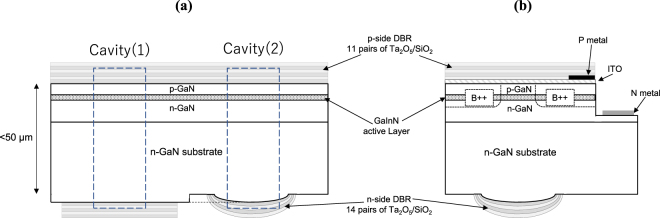
Schematics of the sample structure used in this study. (**a**) GaN-VCSEL used in an optical pumping test where two types of cavities are formed: cavity (1) cladded with two plane mirrors, and cavity (2) with plane and curved mirrors. (**b**) GaN-VCSEL used in current injection testing, where devices with apertures of different diameters (6 and 8 µm) were fabricated on the same substrate.

Experimental results published previously around this idea are limited. A single report on GaN-VCSELs by Park *et al*.^20^ investigated the optical pumping of GaN-VCSELs, where 50-μm-long cavities with plane and curved end mirrors were used. In that case, InGaN quantum wells were grown epitaxially on the (0001) plane of a sapphire template (t = 50 μm), and a curved mirror was fabricated monolithically on the (000-1) plane by using ball-up resin patterns as sacrificial masks during reactive ion etching of sapphire. That study only demonstrated laser operation under optical pumping. The present study investigated lasing under both current injection and optical pumping of VCSELs with a similar structure, where InGaN wells were grown homo-epitaxially on (0001) GaN substrates (see Fig. 2(a,b)). In the present structure, a curved mirror was fabricated on (000-1) GaN rather than sapphire substrates. GaN substrates are known to reduce threading dislocations in quantum wells and suppress the threshold current of GaN-VCSELs as compared to sapphire substrates^12^. Since there were no detailed reports on the quality of curved mirrors fabricated on (000-1) GaN, the present study thoroughly investigated morphological features by atomic force microscopy and confocal laser microscopy. The present study is also the first to confirm the effect of the lateral optical confinement attributed to this structure formed in GaN-based VCSELs. The near-field pattern was measured to observe the effect of lateral optical confinement. This study also investigated the dependence of *J*_*th*_ on aperture size to confirm that curved mirrors enable lateral optical confinement and elimination of the diffraction loss with the present device.

## Results

### Dimension and quality of curved mirrors fabricated on (000-1) GaN

**Figure 3:**
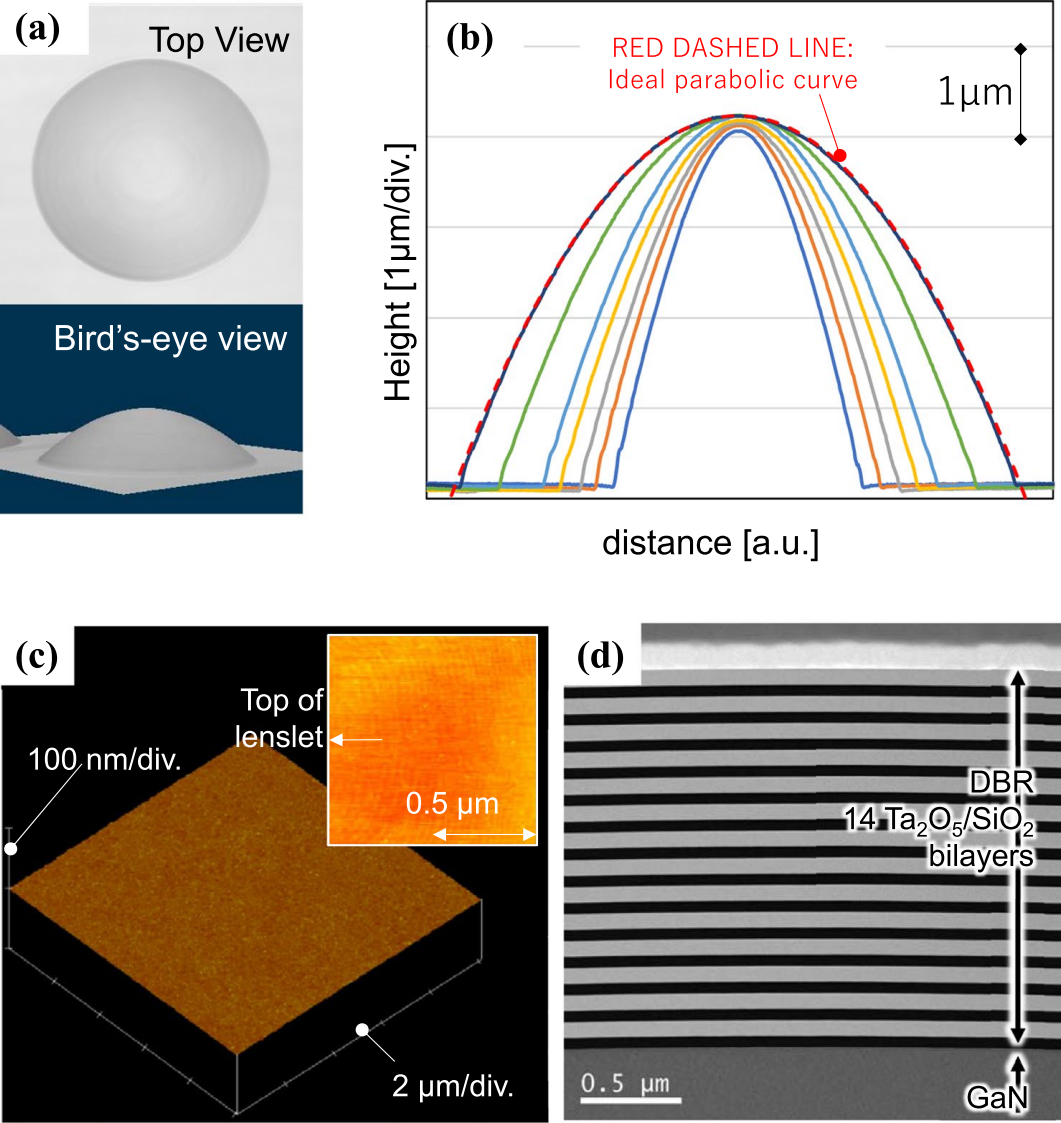
(**a**) Laser scanning confocal microscope images and (**b**) cross-sectional profiles of lenslets (diameter = 55 µm) fabricated on the (000-1) plane of GaN wafer. In panel (**b**), multiple data for different footprint diameters are overlaid. The red dashed line is an ideal parabolic curve fitted to one of the observed data. (**c**) AFM image acquired over top of the lenslets fabricated on the (000-1) plane of a GaN substrate. The inset is a high-resolution image with a 1 µm × 1 um observation area, which shows atomic steps on the surface. (**d**) Cross-sectional TEM image of the curved mirror of the device used for an optical pumping test.

Figure 3(a,b) show the results of laser confocal microscopy performed on a lenslet fabricated on the (000-1) plane of a GaN substrate. The top and bird’s-eye views in Fig. 3(a) both reveal the smooth droplet shape formed. In the cross-sectional analysis (Fig. 3(b)), the radii of curvature of the top of the lenslets were measured to be between 37.7 and 56.6 µm for resin disk diameters ranging from 40 and 56 µm. It is found that the curvature of the top of the lens is positively correlated to the diameter of the footprint of the lenslets determined by the diameter of resin disks used for patterning. Ideal parabolic curves were finely fitted to observed cross-sectional curves. Figure 3(b) shows one example of fitted parabolic curves. An AFM image of the top of the lenslet is shown in Fig. 3(c). The RMS roughness was measured to be 0.2 nm, which is comparable to the value reported for a plane mirror that allows laser operation of GaN-VCSEL^21^. Moreover, the AFM image clearly shows atomic steps at the top of the droplets (see inset in Fig. 3(c)), which indicate that the roughness of this surface is ultimately small for this material system. Figure 3(d) shows a TEM image of the cross-section of the curved mirror of a device. Each DBR layer bends smoothly with the top of the droplets formed on GaN without the introduction of any roughness, forming a perfect curved mirror.

**Figure 4:**
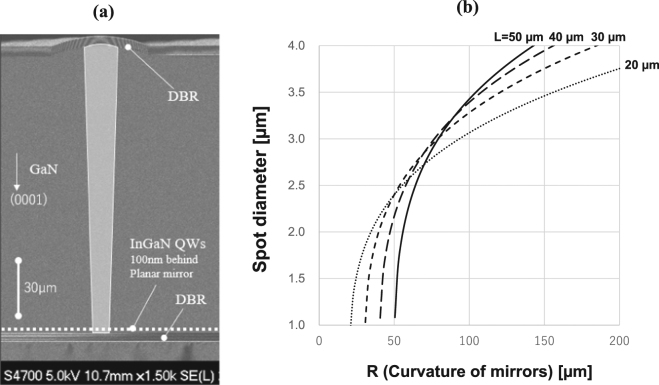
(**a**) Cross-sectional SEM image observed for the device used for the optical pumping test. The light grey shape is a schematic representation of the area for the resonant modes in this device. (**b**) Calculated spot diameter (4σ of the estimated Gaussian curve) on the plane mirror.

Figure 4(a) shows a cross-sectional SEM image of a device used for optical pumping tests. The cavity length of the specimen was measured to be 49.8 μm for this case. Figure 4(b) plots the diameter of the resonant mode (equal to four times σ of the Gaussian profile) on the plane mirrors as a function of radius of curvature for a cavity length from 20 to 50 μm. Along the center of the image in Fig. 4(a) is a schematic of the resonant mode for this cavity, shown as a light gray filled shape. The resonant mode is laterally focused on the plane side, behind which the quantum wells are located only 100 nm away. This indicates that the optical mode is confined to the quantum wells.

### Optical pumping tests for lasing

**Figure 5:**
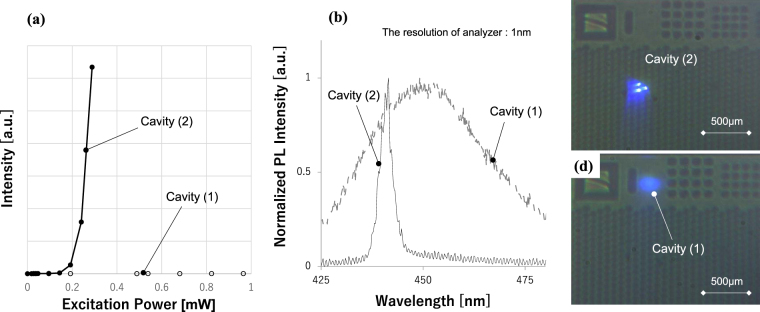
(**a**) Plots for input-output curves for the devices. (**b**) PL spectra of devices under pumping power of 0.3 mW. (**c**) and (**d**) Visual images of specimens under optical pumping for cavity (2) cladded with curved and plane mirrors and cavity (1) with two plane mirrors, respectively.

**Figure 6:**
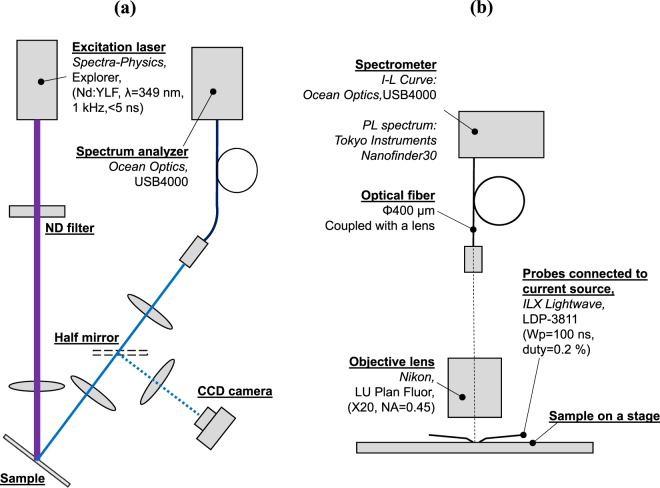
Setups used for (**a**) optical pumping tests and (**b**) current injection test.

Figure 5(a,b) plot the results of optical pumping tests conducted with the setup illustrated in Fig. 6(a) on a device (see schematic in Fig. 2(a)), in which two types of cavities were fabricated: cavity (1) cladded with two plane mirrors and cavity (2) with plane and curved mirrors. In Fig. 5(a), the cavity with a curved mirror exhibits a clear threshold for its optical output at 0.2 mW, while the cavity without a curved mirror does not. In Fig. 5(b), cavities (1) and (2) exhibit emission spectra with a full width at half maximum (FWHM) of 36.5 nm and 3.2 nm, respectively, at a pumping power of 0.3 mW. The resolution limit of the spectrometer, 1 nm, is greater than the longitudinal mode spacing of 0.7 nm, calculated for the cavity length of 49.8 µm. The low resolution of the spectrometer causes the spectrum to have an envelope over multiple longitudinal modes. The results illustrate that about four to five longitudinal modes are selectively amplified above the threshold pumping power for the cavity with a curved mirror. Figure 5(c), which shows a photograph of cavity (2) with a curved mirror under optical pumping, reveals very bright spots. On the other hand, a darker spot is observed in cavity (1) without curved mirrors (see Fig. 5(d)).

### Current Injection test for lasing

**Figure 7:**
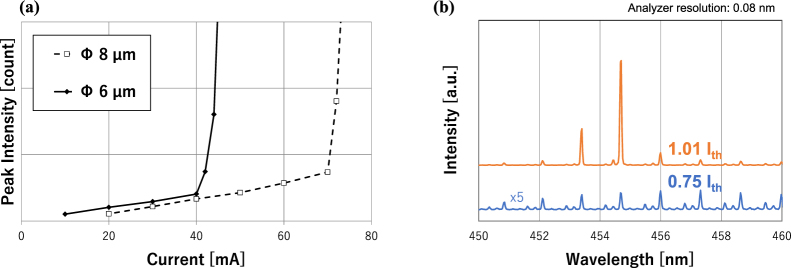
(**a**) I-L curves obtained for devices with an aperture size of 6 and 8 µm and (**b**) spectrum around the threshold current for a typical sample with a 6 µm aperture.

Measurements of I-L, near-field patterns, and output spectra under current injection were made with the device illustrated in Fig. 2(b), using the setup shown in Fig. 6(b). Figure 7(a) shows the I-L curves obtained for two devices with different aperture sizes (6 and 8 μm) fabricated on the same substrate. The threshold currents were 40 and 70 mA for the devices with aperture sizes of 6 and 8 μm, giving *J*_*th*_ values of 139 and 141 kA/cm^2^, respectively. The *J*_*th*_ values were almost the same despite the difference in aperture size. Figure 7(b) is an emission spectrum observed below and above the threshold current for a typical sample with a 6 µm aperture. This clearly shows longitudinal modes and behavior where a few modes were enhanced above the threshold current. The line width was the same as the resolution limit of the analyzer used for the measurement. This illustrates the occurrence of laser operation. The mode spacing of 1.27 nm shown in Fig. 7(b) corresponds to the cavity length of 28.3 µm for the following parameters: n = 2.45, λ = 454 nm, and dn/dλ = −0.001. This length coincides with the value obtained by cross-sectional SEM analysis, 28 µm (data not shown in the figures). Each peak is associated with weaker satellite peaks, which seem to originate from lateral modes of higher orders, as discussed later.

### Measurement of lateral mode size

**Figure 8:**
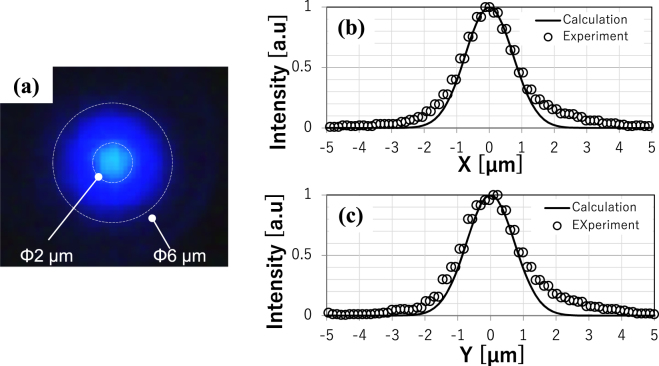
(**a**) Near-field pattern of a device under a current injection of 1.1 I_th_ for an aperture diameter of 6 μm. (**b**) and (**c**) Beam profiles observed along two directions (dots: experimental data, solid lines: theoretical curves calculated using dimensional parameters of the specimen).

To confirm the lateral optical confinement, the lateral mode size was observed by measuring a near-field image. A near-field image was captured by a CCD camera (Fig. 8(a)) under a current injection of 1.1 I_th_ via a band pass filter (450 ± 5 nm). The image shows a single peak pattern spread over a far smaller area within the current aperture diameter of 6 µm. Considering that this image could have been affected by the optical output coming from spontaneous emission, we can say that the lateral mode is confined to within much smaller than 6 µm. Figure 8(b,c) plot spatial intensity distributions measured by the CCD camera and theoretical curves based on the obtained dimensional parameters of the specimen used (cavity length = 28.3 µm, radius of curvature of mirror = 74 µm, standard deviation of lateral mode profile σ = 0.723 µm). The fact that the two plots show a good fit indicates that the resonant mode is confined in the lateral direction, as predicted by Gaussian optics.

## Discussion

In this study, GaN-VCSELs having curved mirrors on one side of their cavities were investigated. Based on the following discussion, we conclude that the long-cavity GaN-VCSELs exhibited laser operation enabled by lateral optical confinement due to the curved mirrors. First, in the optical pumping tests, the cavity with curved mirrors showed a clear threshold in emission power, whereas the cavity with plane mirrors did not (see Fig. 5(a)). The cavity with a curved mirror showed a narrowing of the emission spectrum in the optical pumping test, whereas the cavity with plane mirrors did not (see Fig. 5(b)). This demonstrates the appearance of mode selection and indicates that laser action occurred only in cavities with curved mirrors. The longitudinal mode selection upon threshold was more clearly observed in the current injection test, where each longitudinal mode was visible due to the higher resolution of the spectrometer (see Fig. 7(b)). Those results indicate that the curved mirror improves the quality of the cavity to enable laser operation, even at long cavity lengths up to 49.8 µm for optical pumping and 28.3 µm for the current injection test. Near-field patterns showed that the beam profile can be fitted with a Gaussian with σ = 0.723 µm, the theoretically predicted value for the device used in the experiment, rather than the size of current aperture, 6 μm. Those series of results corroborate the occurrence of lateral optical confinement due to the curved mirror, as predicted by classical Gaussian optics. The current injection tests revealed that *J*_*th*_ was independent of aperture size between 6 and 8 µm. The diffraction of light was enhanced drastically according to the reciprocal of the lateral optical mode size (see Fig. 1(a)). In the absence of lateral optical confinement, the aperture sizes of the present devices should be the primary factor in determining the lateral optical mode size. Thus, a shrinkage of the aperture would lead to a considerable increase in the threshold current density if it were not for lateral optical confinement. The independence of the threshold current density on aperture size further supports the occurrence of lateral optical confinement in the present VCSEL cavities.

Filamentation lasing of GaN-VCSELs was discussed in previous research^8^. In the present research, filamentation is not an appropriate mechanism for the small lateral profile beam used this study based on the following discussion. The diffraction loss of a Gaussian beam with such a small waist (σ = 0.723 µm) and long cavity (L = 28.3 µm) is calculated to reach 50% per round trip without lateral optical confinement (see Fig. 1(a)). This is drastically greater than the expected amplification rate of VCSELs, about 1%^7^. If it were not for lateral optical confinement, lasing would be impossible due to such large diffraction loss.

The authors have selected two topics as future research interests. First, the experimental near-field pattern was spread out slightly wider than predicted by the calculation, and the emission spectrum (Fig. 7(b)) exhibited weak satellite peaks. These are presumably caused by an optical output of higher-order lateral modes^22^. Future research will clarify the relationship between satellite longitudinal modes and lateral modes for the present cavity configuration. Second, the obtained *J*_th_ is much higher than previously reported values. The authors believe one possible factor for this high *J*_th_ is optical absorption of the GaN substrate, which fills most of the cavity. Optical absorption of n-doped GaN is reported to be around 1 cm^−1^ for the doping concentration of the GaN substrate used in the present study (between 1 × 10^−18^ and 1 × 19 cm^−3^)^23^. This gives an optical loss of about 1% per round trip for a 50-µm-long cavity, which is as large as the estimated gain for a typical VCSEL structure^7^. Investigation of the dependence of threshold current on cavity length or Si dopant concentration are potential future research activities.

To the best of our knowledge, the present experimental study marks the achievement of the strongest lateral optical confinement for GaN-based VCSELs to date, which has so far been difficult and has prevented GaN-VCSELs from finding practical application. Moreover, on the basis of classical Gaussian optics, the beam waist of the cavity was controlled by the dimensions both cavity length and radius of curvature of the curved mirror. The dimensions can be easily controlled by standard processes such as photolithography and polishing. Thus, the authors believe that this structure enables further miniaturization of the device to the limit of diffraction, leading to smaller threshold currents for GaN-VCSELs than ever before.

## Methods

### Fabrication and measurement of devices for optical pumping

Figure 2(a,b) illustrate the devices proposed in the present study. The first (Fig. 2(a)) is designed for optical pumping tests. The fabrication process is as follows. Metal-organic chemical vapor deposition (MOCVD) was used to grow four quantum wells (InGaN/GaN MQWs), a p-GaN layer doped with Mg (∼1 × 10^19^ cm^−3^), and a contact layer, to a total thickness of 105 nm, on a (0001)-oriented GaN free-standing substrate doped with Si (between 1 × 10^18^ and 1 × 10^19^ cm^−3^). The final 10 nm-thick layer to be grown is called a contact layer because it is highly doped with Mg (∼1 × 10^20^ cm^−3^) to form an ohmic contact with ITO in the experiment for current injection. The substrates were used as received from the suppliers. A p-side distributed Bragg reflector (DBR) with 11.5 Ta_2_O_5_/SiO_2_ bilayers was deposited on the contact layer. The wafer was lapped to a thickness of less than about 50 μm. Resin disks with diameters ranging from 16 to 80 μm were photolithographed on the lapped face of the GaN wafer, i.e., (000–1). By heating the specimen to 200 °C, the disks were melted into droplets. Reactive ion etching was employed to transfer the surficial shape of the resin droplets onto the GaN substrate by removing them as sacrificial masks, which left a lens-shaped surface on the GaN. An n-side DBR with 14 Ta_2_O_5_/SiO_2_ bilayers was deposited there to form curved mirrors. The disk patterns were designed to form an array with intervals of less than 150 µm, which resulted in the curved mirror arrangement. The length of both cavities, either with a curved mirror on one side or two plane mirrors, is controlled to be the same.

The shape and roughness of the curved mirrors were measured by laser scanning confocal microscopy (Keyence VK-9710) and atomic force microscopy (Bruker Dimension V), respectively. These morphological measurements were conducted directly on the lens-shaped GaN surface before DBR deposition. Scanning electron microscopy (Hitachi Hitech S-4700) and transmission electron microscopy were used to observe the cross-section of the device. Lasing tests were conducted by optical pumping, with the setup illustrated in Fig. 6(a). The excitation source was pulsed at 349 nm (pulse width: <5 ns, frequency: 1 kHz, Spectra Physics Explorer) and injected at 45° to the sample. Its spot size was confined to about 200 µm by inserting a lens before the sample. Because the curved mirrors were arranged with a spatial frequency of less than 150 µm, the irradiated beam must hit one of the cavities regardless of its position on the specimen. The irradiating power was controlled by a variable ND filter set just after the pumping laser. The optical output of the device was measured by the peak power obtained through a spectrometer (Ocean Optics USB4000) via an optical fiber. A CCD camera was used intermittently to observe the sample via a half mirror.

### Fabrication and measurement of devices for current injection

Figure 2(b) shows another structure used to investigate device behavior under current injection. For this experiment, the device was modified by introduction of metal and ITO electrodes to allow current injection. The fabrication process was as follows. MOCVD was used to grow four quantum wells (InGaN/GaN MQWs), a p-GaN layer doped with Mg (∼1 × 10^19^ cm^−3^), and a contact layer doped with Mg (∼1 × 10^20^ cm^−3^), to a total thickness of 105 nm, on a (0001) GaN substrate. A 30-nm-thick ITO layer and a p-side DBR with 11.5 Ta_2_O_5_/SiO_2_ bilayers were deposited on the contact layer by reactive sputtering using a mixture of oxygen and argon gas under the same experimental conditions used in the present authors’ previous studies^7,17,18^. A hole was etched next to the aperture, reaching the n-GaN layer. Two Ti/Pt/Au electrodes were deposited to make contact with the ITO layer and the exposed n-GaN, respectively, to form a current path. A circular current injection region was electrically confined by boron implantation^7,18^, which was arranged on a single wafer and designed to have two different diameters for each device (8 and 6 µm) with an interval of 400 µm. These aperture arrays were placed into contact with individual ITO and Ti/Pt/Au electrodes to form separate emitters. The remaining processing steps followed during curved mirror fabrication were the same as those used to prepare the optical pumping specimen, except that in the former case, the resin disks were patterned precisely opposite the current apertures. The measurement was conducted with specimen of a wafer-level finish, used for tests, I-L curves, spectral measurements, and near-field pattern observation. The current path through these specimens was established via probes touching two Ti/Pt/Au electrodes.

The setup used for I-L and spectral measurements is illustrated in Fig. 6(b). The current source (ILX Lightwave, LDP-3811) was driven under pulsed operation with the conditions Ws = 100 ns, duty = 0.2%. The optical output of the device was determined from the peak count measured by a spectrometer (Ocean Optics, USB4000) via an objective lens (Nikon LU Plan Fluor X20, 0.45). The near-field pattern was measured by a CCD camera via a band pass filter (Asahi Spectra, MX0450, 450 ± 5 nm) placed directly above the sample in order to cut the optical output by spontaneous emission. High-resolution spectra were measured by the detection system of the micro PL measurement system (Tokyo Instruments, Nanofinder30) by using a sample mounted on a can package instead of wafer-style finishing. Scanning electron microscopy (Hitachi Hitech S-4700) was used to observe the cross-section and cavity length of the device.

### Rough estimation of coupling (diffraction) loss of Gaussian beam after a round trip

This section reviews the calculation of the coupling (diffraction) loss between beam before and after a round trip in a VCSEL cavity under the following conditions:

- Cavities are continuously filled with GaN (n = 2.44) without any guiding structures.
- Cavities are cladded with two plane mirrors.
- Beam profiles are ideal Gaussian beams and experience Fraunhofer diffraction.

First, assuming two Gaussian beams having different beam waist radii, the cross-sectional profiles of the electric fields of the beams are expressedphotolithographed on the lapped face of the GaN wafer as follows,

2 $$f(r)=\frac{1}{\sqrt{2\pi {a}^{2}}}\exp (-\frac{{r}^{2}}{2{a}^{2}})\,$$

3 $$g(r)=\frac{1}{\sqrt{2\pi {b}^{2}}}\exp (-\frac{{r}^{2}}{2{b}^{2}}),$$

where a and b are the standard deviation of the two Gaussians before and after a round trip. The coupling efficiency of those two beams is formulated as

4 $$\frac{{\int }^{}fg\,dS}{{\int }^{}{f}^{2}\,dS}=\frac{{\int }_{0}^{\infty }fg\,\,2\pi rdr}{{\int }_{0}^{\infty }{f}^{2}\,2\pi rdr}=\frac{2ab}{{a}^{2}+{b}^{2}}$$

Assuming those beams undergo Fraunhofer diffraction, the ratio of a and b is

5 $$\frac{a}{b}=\sqrt{1+\frac{4{\lambda }^{2}{L}^{2}}{{\pi }^{2}{\omega }_{0}^{4}}},$$

where λ is the lasing wavelength, L is the cavity length and ω_0_ is the beam waist radius of the initial Gaussian beam. Substitution of this formula into the previous one gives the coupling (diffraction) loss after a round trip:

6 $$1-\frac{\sqrt{1+\frac{4{\lambda }^{2}{L}^{2}}{{\pi }^{2}{\omega }_{0}^{4}}}}{1+\frac{2{\lambda }^{2}{L}^{2}}{{\pi }^{2}{\omega }_{0}^{4}}}.$$

### Data availability

This research was conductedas a part of Sony corporation’s researching activity and the all description for the present paper is allowed to be published by Sony corporation. All fund for the present research was supported by Sony corporation.
